# Supplementary material for: Variation of soil physicochemical properties of different vegetation restoration types on subtropical karst area in southern China
Source: PLoS One. 2023 Mar 16;18(3):e0282620. doi: 10.1371/journal.pone.0282620 (PMC10019609; doi:10.1371/journal.pone.0282620)
Supplement: Attachment — Submitted filename: Response to reviewors.docx [file pone.0282620.s002.docx]

**Responses to** **editor and reviewers**

We highly appreciate the valuable comments and suggestions by the editor and reviewers on our manuscript. We have attempted to address each point. Some sentences have been revised or rewritten to improve the English. The following are our detail responses with reference to the order of the comments.

***Part 1:* Point-by-point response to Comments by the editor.**

Editor#: I. Comments

Soil improvement after vegetation restoration is important for rock desertification control. However, there are some vital questions in Methods and Discussion. I suggest the author modified it carefully based on two reviewer's suggestion and re-submit it.

**Re.** First of all, I would like to thank the editor for his high comments on the content of our manuscript and for giving us the opportunity to re-submit. At the same time, we also found that our manuscript still has some shortcomings. Since we received the letter from the editor, we have further modified the content of the manuscript according to the advice of the editor and the reviewers. Due to the large amount of content that needs to be added and the impact of COVID-19, we just finished the manuscript at this time. Below are our responses to comments from editors and reviewers.

We further supplement the methods section of the manuscript with text and pictures and deepen the discussion in the conclusion section. You can see the details in the Methods and Discussion section and figure documentation.

***Part 2:* Point-by-point response to Comments by reviewer 1.**

Reviewer #1: I.

1. There is no detailed information on restoration and planting of 12 vegetation restoration types. What are the physical and chemical properties of the local soil before vegetation restoration? There's no explanation. The field photos of 12 vegetation restoration types should be supplied in the paper.

**Re.** Thanks for the comment! According to your suggestions, we have added detailed information on 12 types of planting to be restored in table1, including the planting time and so on. Since the earliest vegetation restoration time was in 1977, we do not have the physical and chemical properties data of the sample plots at that time. We arranged the rocky desertification wasteland as a CK plot to compare the differences between the physical and chemical properties of the soil after vegetation restoration and without vegetation restoration. And we have added field photos of 12 vegetation restoration types (Figure 2).

2. The determination method of soil physical and chemical properties was not specified in detail.

**Re.** Thank you for your suggestion! We have added ***Experimental methods*** in the **Method** section of the manuscript to explain the determination method of soil physical and chemical properties specified in detail.

3. Line128, the author said that the vegetation coverage in this study area is mainly shrubs and herbs. But what about the tree species in the 12 vegetation restoration types? Why they are not be considered as the local vegetation coverage?

**Re.** We apologize for the ambiguity expression. Here we want to demonstrate the species of herbs and shrubs that grow in the understory during the vegetation restoration. We have revised this sentence. “The shrubs growing in the study area during vegetation restoration are *Camellia sinensis* (L.) O. Ktze., *Eriobotrya deflexa*, *Lindera glauca*, *Alangium chinense*, *Siegesbeckia orientalis* L.

4. Overall, the results of the data analysis in this article were simple and the discussion didn't go far enough.

**Re.** Thank you for your constructive suggestion! Based on the manuscript content and experimental data, we further explored the relationship between soil physical properties and chemical properties of different layers in different vegetation restoration plots through redundancy analysis (RDA), and the results are shown in the ***Results*** section.

We also added the ***Discussion*** section to further discuss vegetation restoration status in rocky desertification areas.

It is suggested that this manuscript should be rejected and resubmitted after major revisions.

**Re.** Thank you for your suggestions and giving us the chance for resubmitting. We have revised it according to the suggestions of the editor and reviewers. We hope that this manuscript can bring theoretical guidance to the vegetation restoration management of rocky desertification areas.

***Part 3:* Point-by-point response to Comments by reviewer 2.**

Reviewer #2:

1. Karst rocky desertification is used to characterize the processes that transform a karst area covered by vegetation and soil into a rocky landscape almost devoid of soil and vegetation. So it is important to assess the impact of ecological restoration measures on soil properties, which will provide scientific references for the future management.

**Re.** Thank you for your suggestions! It is of great significance to evaluate the effect of ecological restoration measures on soil properties. We studied vegetation restoration in the rocky desertification area in the south of Xiangxi, China, and found that there were differences among soils with different vegetation restoration types. It is hoped that the results of our manuscript can provide theoretical guidance for vegetation restoration management in rocky desertification areas.

2. As the title of your paper is about “Variation of soil physicochemical properties of different vegetation restoration types”, it is suggested that moving the results about “Biodiversity and biomass” from the “Results” part to the “Discussion” part and making it clear what is the impacts of “Biodiversity and biomass” on soil, but not discuss “Difference of biomass in different forest management strategy in karst region”, it is another story.

**Re.** Thank you for your constructive suggestion! We have moved the “Biodiversity and biomass” from the “Results” part to the “Discussion” part. The effects of soil biological diversity and biomass on soil physical and chemical properties are further discussed. The specific contents are added in the ***Discussion*** section

2. In the Materials and Methods part and even in the whole paper, it isn’t mentioned what chemical properties of soil are involved in your study. And it is also suggested that telling the readers how you get the data, and the credibility of the data.

**Re.** Sorry, thanks for your advice! We have added ***Experimental methods*** in the **Method** section of the manuscript to explain the information on soil chemical properties of soil and the determination methods we used.
4. As you choose “a space-for-time substitution approach”, the comparability of your plots should be considered, so it is suggested that telling the reader the planting time or the beginning of restoration of the 12 plots.

**Re.** Thanks for your useful advice! According to your suggestions, we have added detailed information on 12 types of planting to be restored in table 1, including the planting time and so on. And we have added field photos of 12 vegetation restoration types (Figure 2).
5. And the language should be revised carefully.

**Re.** Thanks for the comment! We have revised and polished the whole manuscript.
