# Supplementary material for: Variation of soil physicochemical properties of different vegetation restoration types on subtropical karst area in southern China
Source: PLoS One. 2023 Mar 16;18(3):e0282620. doi: 10.1371/journal.pone.0282620 (PMC10019609; doi:10.1371/journal.pone.0282620)
Supplement: Attachment — Submitted filename: PONE-D-22-29404 (review report).docx [file pone.0282620.s003.docx]

PONE-D-22-29404:

Variation of soil physicochemical properties of different vegetation restoration types on subtropical karst area in southern China

Soil physical properties in the topsoil samples from the forest management areas were significantly higher than uncultivated lands (P < 43 0.05).

(3) Redundancy analysis showed that the soil chemical content differed significantly among the types of forest vegetation restoration and different soil layers.

Among the nutrients analyzed, Mg, Zn and K were the main factors affecting soil properties in the rocky desertification areas.

Therefore, our results recommend planting the broadleaved deciduous species and coniferous species as the preferred tree species to enhance the soil fertility and water conservation functions, especially in subtropical karst area ecosystems, which provided for making scientific forest restoration 50 management in karst region.

LN 153-154: Soil physical properties were measured by the cutting ring method. Q#: What is cutting ring method? There is no references? Which parameters were analysed by Cutting ring method?

LN 159-160: The HNO3-HCl-HClO4 atomic absorption spectrometry method was used to measure soil Ca and Mg. The contents of P, K, Na, Fe, Cu, Mn, Pb, Cd and Zn in soil were determined by ICP-AES.

Q#: Experimental methods – are extremely poor, cannot be accepted for any international journal.

Recommendation: REJECT.
