# Supplementary material for: Variation of soil physicochemical properties of different vegetation restoration types on subtropical karst area in southern China
Source: PLoS One. 2023 Mar 16;18(3):e0282620. doi: 10.1371/journal.pone.0282620 (PMC10019609; doi:10.1371/journal.pone.0282620)
Supplement: Attachment — Submitted filename: Response to Reviewers.docx [file pone.0282620.s004.docx]

**Responses to** **editor and reviewers**

We highly appreciate the valuable comments and suggestions by reviewers on our manuscript. We made further modifications according to the suggestions of the reviewers and have attempted to address each point. The following are our details responses with reference to the order of the comments.

***Part 1:* Response to Comments by the editor.**

PONE-D-22-29404R1
Variation of soil physicochemical properties of different vegetation restoration types on subtropical karst area in southern China
PLOS ONE

Dear Dr. Ninghua,

Thank you for submitting your manuscript to PLOS ONE. After careful consideration, we feel that it has merit but does not fully meet PLOS ONE’s publication criteria as it currently stands. Therefore, we invite you to submit a revised version of the manuscript that addresses the points raised during the review process.

==============================
ACADEMIC EDITOR: In my opinion, the manuscript is much improved compared to the first draft. But as one reviewer pointed out, some problems still exist, the authors should respond to the comments and try to improve the quality of the manuscript.

**Re.** Thanks for your positive comments on our manuscript. The suggestions put forward by the reviewers are very helpful for the further optimization of our paper. We make point-to-point responses based on the comments of reviewers.

***Part 1:* Point-by-point response to Comments by the reviewer.**

**Comments to the Author**

1. If the authors have adequately addressed your comments raised in a previous round of review and you feel that this manuscript is now acceptable for publication, you may indicate that here to bypass the “Comments to the Author” section, enter your conflict of interest statement in the “Confidential to Editor” section, and submit your "Accept" recommendation.

Reviewer #2: (No Response)

Reviewer #4: All comments have been addressed

Re. Thanks for your comments

2. Is the manuscript technically sound, and do the data support the conclusions?
The manuscript must describe a technically sound piece of scientific research with data that supports the conclusions. Experiments must have been conducted rigorously, with appropriate controls, replication, and sample sizes. The conclusions must be drawn appropriately based on the data presented.

Reviewer #2: No

Reviewer #4: Yes

Re. Thanks for your comments

3. Has the statistical analysis been performed appropriately and rigorously?

Reviewer #2: No

Reviewer #4: Yes

Re. Thanks for your comments

4. Have the authors made all data underlying the findings in their manuscript fully available?

The [PLOS Data policy](http://www.plosone.org/static/policies.action#sharing) requires authors to make all data underlying the findings described in their manuscript fully available without restriction, with rare exception (please refer to the Data Availability Statement in the manuscript PDF file). The data should be provided as part of the manuscript or its supporting information, or deposited to a public repository. For example, in addition to summary statistics, the data points behind means, medians and variance measures should be available. If there are restrictions on publicly sharing data—e.g. participant privacy or use of data from a third party—those must be specified.

Reviewer #2: No

Reviewer #4: (No Response)

Re. Thanks for your comments

5. Is the manuscript presented in an intelligible fashion and written in standard English?

PLOS ONE does not copyedit accepted manuscripts, so the language in submitted articles must be clear, correct, and unambiguous. Any typographical or grammatical errors should be corrected at revision, so please note any specific errors here.

Reviewer #2: Yes

Reviewer #4: Yes

Re. Thanks for your comments

6. Review Comments to the Author

Please use the space provided to explain your answers to the questions above. You may also include additional comments for the author, including concerns about dual publication, research ethics, or publication ethics. (Please upload your review as an attachment if it exceeds 20,000 characters)

Reviewer #2:

1.The method that the authors dealt with the data is not right. "In the present study, we divided 12 different tree species into three different forest vegetation types (CF, EB, BD). And we averaged the soil physical and chemical properties of three different forest vegetation types respectively for further data processing”.

Re. Sorry for the wrong expression. We have revised this paragraph as “In this study, soil physical and chemical properties of 12 tree species were treated on average (groups of repetitions), and then 12 different tree species were divided into three different forest vegetation types (CF, EB, BD) for further data processing.” Hope the revised version could be more clear.

2.About the chemical properties: Al, Ca, Cd, Cu, Fe, K, Mg, Mn, Na, P, Pb, and Zn, the relationship of the vegetation and these elements is still not clear in the study.

Re. Sorry. We have added an analysis of the relationship between the vegetation and these elements, which is shown in the part “Effects of stand type characteristics on soil basic physical and chemical properties”.

3.There is still something wrong in the article, for example, "The soil chemical and physical properties and plant diversity of 10 vegetation types in Northwest Hunan were analysed."

Re. Sorry for the mistakes. We have rechecked the manuscript and made further modifications.
4.The conclusion is not sound because all the analyzing is based on the three group CF, EB, BD, but not "Liriodendron chinense (Hemsl.) Sarg".

Re. Thanks for your valuable comments. We have revised this part as “For these characteristics, the broadleaved deciduous forest may be the most suitable forest type in forest restoration management in the subtropical karst area of southern China.”

Reviewer #4: The manuscript is much improved compared to the first draft. The authors did well to add experimental and analytical details, improving the clarity of their writing. I think the manuscript is close to publication-ready.

Re. Thanks for your positive comments.
